# Analysis of Aggregation Delay for Multisource Sensor Data with On-Off Traffic Pattern in Wireless Body Area Networks

**DOI:** 10.3390/s16101622

**Published:** 2016-09-30

**Authors:** Un-Ha Kim, Eutteum Kong, Hyun-Ho Choi, Jung-Ryun Lee

**Affiliations:** 1 School of the Electrical Engineering, Chung-Ang University, 84 Heukseok-ro, Dongjak-gu, Seoul 06974, Korea; kimhr0703@cau.ac.kr (U.-H.K.); etkong@cau.ac.kr (E.K.); 2 Department of Electrical, Electronic and Control Engineering, Hankyong National University, 327 Chungang-ro, Anseong 17579, Korea

**Keywords:** data aggregation, aggregation delay, delay analysis, body area networks

## Abstract

Data aggregation plays an important role to improve the transmission efficiency in wireless body area networks (WBANs); however, it inherently induces additional aggregation delay. Therefore, the effect of packet aggregation on WBAN applications, which are vulnerable to delay, must be analyzed rigorously. In this paper, we analyze the packet aggregation delay for multisource sensor data with an on-off traffic pattern in WBANs. Considering two operational parameters of the aggregation threshold and aggregation timer, we calculate the probability that a packet aggregation occurs during a unit time and then derive the average aggregation delay in closed-form. The analysis results show that the aggregation delay increases as the aggregation timer or aggregation threshold increases, but is bounded below a certain level according to the number of active sensors and their on-off traffic attribute. This implies that the data aggregation technique can maximize the transmission efficiency while satisfying a given delay requirement in the WBAN system.

## 1. Introduction

The wireless body area network (WBAN) is a wireless network of wearable sensing, computing and communicating devices. In the WBAN, the wearable devices communicate with one another in the proximity of a human body and also connect to the Internet through gateway devices (e.g., a cellular phone) [1,2]. With such a kind of network connectivity, the WBAN provides various interdisciplinary applications, including remote medical diagnosis, interactive gaming, military applications, etc. The wearable devices monitor the human’s status and transmit all sensing data in real time or trigger alarms in abnormal conditions to the healthcare server. In an interactive game, sensors feed back information about actual body movements of game players to the corresponding gaming console to provide entertainment experiences. Furthermore, WBANs can be used to connect soldiers in a battlefield and report their activities to the commander. In such applications, body sensors provide sensing data to the body area aggregator, which is central to manage body events. The aggregator node gathers all sensing data and forwards them to other infrastructures via wireless backhaul after a pre-processing, such as data fusion or data aggregation, in order to enhance the transmission efficiency or reduce the energy consumption [3,4].

The use of data aggregation in WBANs can increase the transmission efficiency by combining small multiple sensor data into a single transmission frame, thereby reducing the overhead associated with each transmission [5]. Data aggregation is useful in situations where each transmission frame has a significant overhead (i.e., preambles, headers, trailers, etc.) or where the expected size of sensing information is quite small compared to the maximum frame size. The data aggregation reduces the overhead and, so, enhances the transmission efficiency, but causes an additional delay to wait for sufficient sensor data to be aggregated. That is, there commonly exists a tradeoff between the transmission efficiency and the aggregation delay in the use of data aggregation [6]. Since the aggregation delay directly affects the end-to-end delay of sensing data, the analysis of aggregation delay is very important to provide the quality of services (QoS) for the applications of WBAN.

Research about data aggregation was originated from a wired network, and the first packet aggregation methods have been proposed to increase the transmission efficiency in Ethernet [7,8]. In a wireless network, studies on packet aggregation have been conducted to fully utilize the limited bandwidth of the air interface [9]. The packet aggregation for real-time traffic with a strict QoS constraint has been investigated by considering the unstable wireless link [10]. Moreover, the packet aggregation delay has been evaluated, and its effect on the end-to-end delay of voice traffic has been shown [11]. In addition, the so-called holding time aggregation method has been proposed for real-time traffic, in which a packet holding time was estimated to ensure the QoS, such as end-to-end delay and jitter [12]. Similarly, an aggregation algorithm to satisfy the QoS in WBAN networks has been proposed by introducing the critical delay as a parameter, in order to serve packets taking into account their priorities and classifying them into an aggregated frame [13]. Various aspects of QoS with a focus on WBAN have been presented [14]. This study informs that the data fusion and aggregation seriously influence the overall QoS in WBANs. According to different medium access control protocols, realistic experiments have been performed, and then, a data aggregation strategy has been proposed [15]. These results show that the data aggregation significantly improves the reliability and energy consumption in WBANs. On the other hand, an energy-efficient aggregation and reliable communication protocol for WBANs has been proposed [16]. Therein, the aggregator nodes were initially chosen based on the nodes’ connectivity, and the network coding was applied to enhance both the energy efficiency and the reliability of data transmission. An energy consumption-balanced method of data aggregation for wearable sensor systems has been introduced [17]. This method combines a query algorithm to construct a routing tree and a distributed data aggregation algorithm in order to prolong the lifetime of wearable sensor networks.

Previous studies have mostly considered a constant bit rate traffic that does not have an on-off traffic pattern. However, some WBAN applications generate non-real-time traffic, as well as real-time traffic, so there is a need to consider a variable bit rate traffic with an on-off traffic pattern. In this paper, we consider the aggregation of various sensor data with an on-off traffic pattern from the practical perspective. To this end, we consider the aggregation threshold and the aggregation timer as the operational parameters of the aggregation process. The aggregation threshold refers to the maximum number of packets aggregated into a single frame, which reflects the fact that the available bandwidth of wireless backhaul is limited [18]. The aggregation timer refers to the maximum allowable time to wait for packets before transmission, which limits the maximum queueing delay at the buffer of the aggregator, especially when the sensor data are generated sparsely. In addition to such new considerations, our main contribution is to derive a closed-form expression of aggregation delay, as an important performance metric of the packet aggregation process, considering multisource sensor data with an on-off traffic pattern. This analytic framework is quite distinct from previous studies and allows one to easily identify the performance tendency of aggregation delay according to various operational parameters. Consequently, this study can contribute to providing a theoretical basis for further research on the performance evaluation in WBANs.

The rest of this paper is organized as follows. Section 2 describes the system model and notations used for analysis. Section 3 first analyzes the aggregation probability numerically, and then, Section 4 calculates the average aggregation delay in closed-form. Section 5 verifies the numerical analysis compared with the simulation and shows the tendency of aggregation delay according to various parameters. Finally, Section 6 provides the concluding remarks of this study.

## 2. System Description

**Figure 1:**
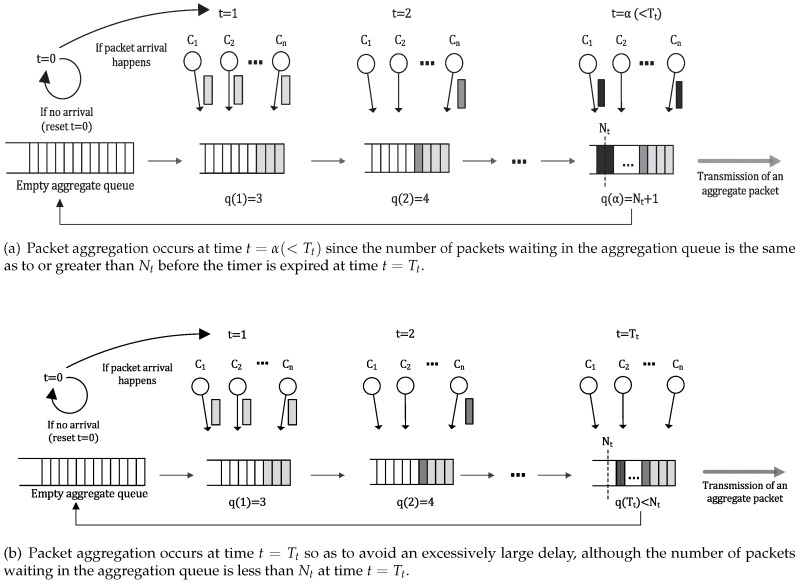
Schematic representation of the packet aggregation process.

Figure 1 illustrates a schematic representation of the considered packet aggregation process. Suppose that the considered WBAN has *C* traffic sources (i.e., active sensors). All of the data generated from these sensors are aggregated at a central gateway node (i.e., body area aggregator) and forwarded to the Internet server via wireless backhaul. The aggregator stores the incoming sensor data in an aggregate queue. If the number of queued packets is the same as or greater than the predetermined packet aggregation threshold, the queued packets are immediately transmitted as an aggregate packet. We denote this predetermined packet aggregation threshold as $N_{t}$. In reality, the aggregation threshold is required due to the limited bandwidth of wireless backhaul.

Because of the on-off characteristic of traffic, the variable waiting (or queueing) delay occurs during the aggregation process. If the time taken for the number of queued packets to be the same as or greater than $N_{t}$ is excessively long, the queueing delay at the aggregator significantly increases, and the QoS deteriorates. To prevent this excessive queueing delay, the aggregator transmits an aggregated frame when the aggregation timer expires, although the number of packets in a queue is less than $N_{t}$. We denote the value of the packet aggregation timer as $T_{t}$.

Most codecs use various silence suppression or compression techniques to prevent the unnecessary generation of packets during the no information period, thus avoiding the waste of limited bandwidth [19]. The on-off traffic attribute of various applications is modeled by some statistical analysis [20]. For example, a voice detector generates packets for a period of 60 percent of the entire session on average, and no packet is generated during the rest period. Thus, the traffic with the on-off period is modeled as a successive Bernoulli trial based on a discrete time index *t* with a probability of $P_{a}$, which is the probability that a sensor generates a packet during a given time period [21]. The symbols used for analysis and their definitions are summarized in Table 1.

## 3. Analysis of the Aggregation Probability

In this section, we first try to calculate the probability that the packet aggregation occurs at time *t* (i.e., the aggregated frame is transmitted at time *t*). We call this probability the aggregation probability in this study.

Suppose that the first packet arrival at the aggregator occurs during the time interval [0,1]. The probability that *i* packets arrive in the queue during the unit time, $\alpha_{i}$, is given by:

(1) αi=CiPai(1−Pa)C−ifor0≤i≤C.

Let $PA_{N_{t}}^{T_{t}}$ be the random variable denoting the time when the packet aggregation process occurs, given that the values of the aggregation timer and aggregation threshold are $T_{t}$ and $N_{t}$, respectively. Let $P_{T_{t}}$ and $P_{N_{t}}$ be the probabilities that the queued packets are aggregated by the expiration of the aggregation timer and by the number of the queued packets being the same as or greater than the packet aggregate counter, respectively. We then have the following equation:

(2) $$\sum_{t=1}^{T_{t}}P\left[PA_{N_{t}}^{T_{t}}=t\right]=P_{N_{t}}+P_{T_{t}}=1.$$

Let $p_{i}\left(t\right)$ be the probability that the number of queued packets is *i* at time *t* given that the aggregation timer starts at Time 0. Then, there should be at least one packet arrival during the time interval [0,1]. From the definition of q(t), $P_{T_{t}}$ is given by:

(3) $$\begin{array}{cc} P_{T_{t}} & =P[q\left(T_{t}\right)<N_{t}|q\left(1\right)>0]\\ & =\sum_{i=1}^{N_{t}-1}P\left[q\left(T_{t}\right)=i|q\left(1\right)>0\right]=\sum_{i=1}^{N_{t}-1}p_{i}\left(T_{t}\right). \end{array}$$

Let $\tilde{p_{i}}\left(\tau \right)$ be the probability that there are *i* queued packets during the time interval [t,t+τ], that is $\tilde{p_{i}}\left(\tau \right)=P\left[q\left(\tau \right)=i\right]$. Then, because of the independent packet arrivals at the aggregator, $\tilde{p_{i}}\left(1\right)$ is time-homogeneous and is given by:

(4) $$\tilde{p_{i}}\left(1\right)=p_{i}\left(1\right)=P\left[q\left(1\right)=i\right]=\left\{\begin{array}{cc} \alpha_{i}, & 0\leq i\leq C\\ 0, & \mathrm{otherwise} \end{array}.\right.$$

If there are *i* queued packets during the time interval [t,t+τ], this implies that i−k packets arrive in the queue during the time interval of [t,t+1] and *k* packets arrive in the queue during the remaining time interval [t+1,t+τ], where *k* varies from zero to *i*. Therefore, we have:

(5) $$\tilde{p_{i}}\left(\tau \right)=\sum_{k=0}^{i}\alpha_{i-k}\tilde{p_{k}}\left(\tau -1\right).$$

Let $\tilde{P}\left(t\right)={\left[\tilde{p_{0}}\left(t\right),\tilde{p_{1}}\left(t\right),\ldots ,{\tilde{p}}_{N_{t}-1}\left(t\right)\right]}^{T}$. From Equations (4) and (5), we can derive the following linear system:

(6) $$\tilde{P}\left(t\right)=A\cdot \tilde{P}\left(t-1\right)=A^{t-1}\cdot \tilde{P}\left(1\right)\;\;\mathrm{for}\;\;t\geq 2$$

where A is a $N_{t}\times N_{t}$ lower triangular matrix expressed as:

(7) $$A=\left\{\alpha_{i,j}\right\}=\left[\begin{array}{lllll} \alpha_{0} & 0 & 0 & \cdots & 0\\ \alpha_{1} & \alpha_{0} & 0 & \cdots & 0\\ \alpha_{2} & \alpha_{1} & \alpha_{0} & \cdots & 0\\ \vdots & \vdots & \vdots & \ddots & \vdots \\ \alpha_{N_{t}-1} & \alpha_{N_{t}-2} & \alpha_{N_{t}-3} & \cdots & \alpha_{0} \end{array}\right]$$

and $\tilde{P}\left(1\right)={\left[\alpha_{0},\alpha_{1},\ldots ,\alpha_{N_{t}-1}\right]}^{T}$. Let $\left\{\beta_{i,j}^{\left(t\right)}\right\}=B^{\left(t\right)}:=A^{t}$. Then, matrix $B^{\left(t\right)}$ inherits the properties of matrix A, that is $B^{\left(t\right)}$ is also a $N_{t}\times N_{t}$ lower triangular matrix whose elements on the main diagonal are the same as $\beta_{0}^{\left(t\right)}$ and the elements on the *k*-th diagonal below the main diagonal are the same as $\beta_{k}^{\left(t\right)}$. From Equation (6), we have:

(8) $$\begin{array}{cc} \tilde{P}\left(t\right) & =\left[\begin{array}{lllll} \beta_{0}^{\left(t-1\right)} & 0 & 0 & \cdots & 0\\ \beta_{1}^{\left(t-1\right)} & \beta_{0}^{\left(t-1\right)} & 0 & \cdots & 0\\ \beta_{2}^{\left(t-1\right)} & \beta_{1}^{\left(t-1\right)} & \beta_{0}^{\left(t-1\right)} & \cdots & 0\\ \vdots & \vdots & \vdots & \ddots & \vdots \\ \beta_{N_{t}-1}^{\left(t-1\right)} & \beta_{N_{t}-2}^{\left(t-1\right)} & \beta_{N_{t}-3}^{\left(t-1\right)} & \cdots & \beta_{0}^{\left(t-1\right)} \end{array}\right]\tilde{P}\left(1\right)\\ & =\left[\begin{array}{c} \beta_{0}^{\left(t-1\right)}\alpha_{0}\\ \beta_{1}^{\left(t-1\right)}\alpha_{0}+\beta_{0}^{\left(t-1\right)}\alpha_{1}\\ \vdots \\ \sum_{i=0}^{N_{t}-1}\beta_{N_{t}-1-i}^{\left(t-1\right)}\alpha_{i} \end{array}\right]=\left[\begin{array}{c} \beta_{0}^{\left(t\right)}\\ \beta_{1}^{\left(t\right)}\\ \vdots \\ \beta_{N_{t}-1}^{\left(t\right)} \end{array}\right], \end{array}$$

where the last equality comes from the following equation:

(9) $$\begin{array}{cc} B^{\left(t\right)} & =B^{\left(t-1\right)}A\\ & =\left[\begin{array}{lllll} \beta_{0}^{\left(t-1\right)} & 0 & 0 & \cdots & 0\\ \beta_{1}^{\left(t-1\right)} & \beta_{0}^{\left(t-1\right)} & 0 & \cdots & 0\\ \beta_{2}^{\left(t-1\right)} & \beta_{1}^{\left(t-1\right)} & \beta_{0}^{\left(t-1\right)} & \cdots & 0\\ \vdots & \vdots & \vdots & \ddots & \vdots \\ \beta_{N_{t}-1}^{\left(t-1\right)} & \beta_{N_{t}-2}^{\left(t-1\right)} & \beta_{N_{t}-3}^{\left(t-1\right)} & \cdots & \beta_{0}^{\left(t-1\right)} \end{array}\right]\cdot A\\ & =\left[\begin{array}{llll} \beta_{0}^{\left(t-1\right)}\alpha_{0} & 0 & \cdots & 0\\ \beta_{1}^{\left(t-1\right)}\alpha_{0}+\beta_{0}^{\left(t-1\right)}\alpha_{1} & \beta_{0}^{\left(t-1\right)}\alpha_{0} & \cdots & 0\\ \vdots & \vdots & \ddots & \vdots \\ \sum_{i=0}^{N_{t}-1}\beta_{N_{t}-1-i}^{\left(t-1\right)}\alpha_{i} & \cdots & \cdots & \beta_{0}^{\left(t-1\right)} \end{array}\right] \end{array}$$

The relation between ${\tilde{p}}_{i}\left(t\right)$ and $p_{i}\left(t\right)$ for t≥2 is given by:

(10) $$\begin{array}{cc} p_{i}\left(t\right) & =P\left[q\left(t\right)=i|q\left(1\right)>0\right]=\frac{P[q(t)=i,q(1)>0]}{P[q(1)>0]}\\ & =\frac{1}{1-\alpha_{0}}\sum_{k=1}^{C}P\left[q\left(t\right)=i,q\left(1\right)=k\right]P\left[q\left(1\right)=k\right]\\ & =\frac{1}{1-\alpha_{0}}\sum_{k=1}^{C}\alpha_{k}P\left[q\left(t-1\right)=i-k\right]\\ & =\frac{1}{1-\alpha_{0}}\sum_{k=1}^{C}\alpha_{k}{\tilde{p}}_{i-k}\left(t-1\right)=\frac{1}{1-\alpha_{0}}\left({\tilde{p}}_{i}\left(t\right)-{\tilde{p}}_{i}\left(t-1\right)\alpha_{0}\right) \end{array}$$

where the last equality comes from ${\tilde{p}}_{i}\left(t\right)=\sum_{k=0}^{C}\alpha_{k}{\tilde{p}}_{i-k}\left(t-1\right)$. From Equations (3), (9) and (10), we have:

(11) $$\begin{array}{cc} P_{T_{t}} & =\sum_{i=1}^{N_{t}-1}p_{i}\left(T_{t}\right)\\ & =\frac{1}{1-\alpha_{0}}\sum_{i=1}^{N_{t}-1}\left(\tilde{p_{i}}\left(T_{t}\right)-\tilde{p_{i}}\left(T_{t}-1\right)\alpha_{0}\right)\\ & =\frac{1}{1-\alpha_{0}}\sum_{i=1}^{N_{t}-1}\left(\beta_{i}^{\left(T_{t}\right)}-\beta_{i}^{\left(T_{t}-1\right)}\alpha_{0}\right). \end{array}$$

From Equations (2) and (11), $P_{N_{t}}$ is given by:

(12) $$P_{N_{t}}=1-\frac{1}{1-\alpha_{0}}\sum_{i=0}^{N_{t}-1}\left(\beta_{i}^{\left(T_{t}\right)}-\beta_{i}^{\left(T_{t}-1\right)}\alpha_{0}\right).$$

The event that the packet aggregation process occurs at time $T_{t}$ means that the number of queued packets at time $T_{t}-1$ is less than $N_{t}$; thus, $P[PA_{N_{t}}^{T_{t}}=T_{t}]$ for $T_{t}>2$ is calculated by:

(13) $$\begin{array}{cc} P[PA_{N_{t}}^{T_{t}}=T_{t}] & =P[q\left(T_{t}-1\right)<N_{t}|q\left(1\right)>0]\\ & =\sum_{i=1}^{N_{t}-1}p_{i}\left(T_{t}-1\right)\\ & =\frac{1}{1-\alpha_{0}}\sum_{i=1}^{N_{t}-1}\left(\beta_{i}^{\left(T_{t}-1\right)}-\beta_{i}^{\left(T_{t}-2\right)}\alpha_{0}\right). \end{array}$$

Note that the event of $PA_{N_{t}}^{T_{t}}=T_{t}$ does not always mean that the packet aggregation process is triggered by the expiry of the aggregation timer. Suppose that there are four queued packets in an aggregate queue until the elapsed time is 9 with the assumptions that $T_{t}=10$ and $N_{t}=5$. If there are one or more packet arrivals in an aggregate queue at Time 10, the packet aggregate process is triggered by both the aggregation threshold and the expiry of the aggregation timer. Otherwise, if there is no packet arrival at Time 10, the packet aggregation process is activated solely by the aggregation timer.

From 0≤q(1)≤C and 0≤q(i+1)−q(i)≤C for all i>1, the aggregation probabilities at Times 1 and 2 are manually calculated, respectively, as given by:

(14) $$\begin{array}{cc} P[PA_{N_{t}}^{T_{t}}=1] & =P[N_{t}\leq q\left(1\right)\leq C|q\left(1\right)>0]\\ & =\frac{1}{1-\alpha_{0}}\sum_{i=N_{t}}^{C}\alpha_{i}\;\mathrm{for}\;C\geq N_{t}, \end{array}$$

(15) $$\begin{array}{cc} P & \left[PA_{N_{t}}^{T_{t}}=2\right]=P\left[q\left(1\right)<\min\left\{C,N_{t}-1\right\},q\left(1\right)+q\left(2\right)\geq N_{t}|q\left(1\right)>t\right]\\ & =P[N_{t}\;-\;C\leq q\left(1\right)\leq \min\left\{C,N_{t}\;-\;1\right\},N_{t}\;-\;q\left(1\right)\leq q\left(2\right)\leq \min\left\{C,N_{t}\;+\;C\;-\;1\;-\;q\left(1\right)\right\}|q\left(1\right)\;>\;0]\\ & =\frac{1}{1-\alpha_{0}}\sum_{i=N_{t}-C}^{\min\{C,N_{t}-1\}}\sum_{j=N_{t}-i}^{\min\{C,N_{t}+C-1-i\}}\alpha_{i}\alpha_{j}\;\;\;\mathrm{for}\;\;C\geq \frac{N_{t}}{2}. \end{array}$$

To determine the aggregation probability at an arbitrary time t>2, we make the following proposition.

**Proposition:**  *For t>0, we have:*

$$P\left[PA_{N_{t}}^{t+1}=t\right]=P\left[PA_{N_{t}}^{t}=t\right]-P\left[PA_{N_{t}}^{t+1}=t+1\right].$$

**Proof.**  Suppose that the packet aggregation process is triggered at time *k* by the aggregation threshold. Then, a value of the aggregation timer greater than *k* has no effect on the aggregation probability, that is,

(16) $$P\left[PA_{N_{t}}^{a}=k\right]=P\left[PA_{N_{t}}^{b}=k\right]\;\mathrm{for}\;\mathrm{all}\;a,b>k.$$

Then, we have:

$$\begin{array}{cc} 1= & \sum_{k=1}^{t+1}P\left[PA_{N_{t}}^{t+1}=k\right]\\ = & \sum_{k=1}^{t-1}P\left[PA_{N_{t}}^{t+1}=k\right]+P\left[PA_{N_{t}}^{t+1}=t+1\right]+P\left[PA_{N_{t}}^{t+1}=t\right]\\ = & \sum_{k=1}^{t-1}P\left[PA_{N_{t}}^{t}=k\right]+P\left[PA_{N_{t}}^{t+1}=t+1\right]+P\left[PA_{N_{t}}^{t+1}=t\right]\\ = & \;1-P\left[PA_{N_{t}}^{t}=t\right]+P\left[PA_{N_{t}}^{t+1}=t+1\right]+P\left[PA_{N_{t}}^{t+1}=t\right], \end{array}$$

which ends the proof. ☐

From Equations (13) and (16) and the above Proposition, the probability that the packet aggregation process occurs at time $t<T_{t}$ is given by:

(17) $$\begin{array}{cc} P[PA_{N_{t}}^{T_{t}}=t] & =P[PA_{N_{t}}^{t+1}=t]\\ & =P\left[PA_{N_{t}}^{t}=t\right]-P\left[PA_{N_{t}}^{t+1}=t+1\right]\\ & =\frac{1}{1-\alpha_{0}}\;\left(\sum_{i=1}^{N_{t}-1}\left(\beta_{i}^{\left(t-1\right)}-\beta_{i}^{\left(t-2\right)}\alpha_{0}\right)-\;\sum_{i=1}^{N_{t}-1}\left(\beta_{i}^{\left(t\right)}-\beta_{i}^{\left(t-1\right)}\alpha_{0}\right)\right)\\ & =\frac{1}{1-\alpha_{0}}\sum_{i=1}^{N_{t}-1}\left(\left(1+\alpha_{0}\right)\beta_{i}^{\left(t-1\right)}-\beta_{i}^{\left(t\right)}-\alpha_{0}\beta_{i}^{\left(t-2\right)}\right). \end{array}$$

From Equations (13)–(15) and (17), the aggregation probability at time *t* is obtained by:

(18) $$\begin{array}{cc} P & \left[PA_{N_{t}}^{T_{t}}=t\right]=\left\{\begin{array}{cc} \frac{1}{1-\alpha_{0}}\sum_{i=N_{t}}^{C}\alpha_{i}, & t=1\\ \frac{1}{1-\alpha_{0}}\sum_{i=N_{t}-C}^{\min\{C,N_{t}-1\}}\sum_{j=N_{t}-i}^{\min\{C,N_{t}+C-1-i\}}\alpha_{i}\alpha_{j}, & t=2\\ \frac{1}{1-\alpha_{0}}\sum_{i=1}^{N_{t}-1}\left(\left(1+\alpha_{0}\right)\beta_{i}^{\left(t-1\right)}-\beta_{i}^{\left(t\right)}-\alpha_{0}\beta_{i}^{\left(t-2\right)}\right), & 2\leq t<T_{t}\\ \frac{1}{1-\alpha_{0}}\sum_{i=1}^{N_{t}-1}\left(\beta_{i}^{\left(T_{t}-1\right)}-\beta_{i}^{\left(T_{t}-2\right)}\alpha_{0}\right), & t=T_{t}. \end{array}\right. \end{array}$$

## 4. Analysis of the Average Aggregation Delay

The aggregation delay is defined as the time difference between the time when the packet aggregation occurs and the time when the packet arrives at the aggregate queue. In our work, a packet *m* that arrives at the aggregation queue during $S_{t-x}$ is regarded to have arrived at the aggregation queue at time t−x precisely; the aggregation delay of packet *m* is therefore *x* given that the packet aggregation occurs at time *t*. Let *X* be a random variable denoting this aggregation delay of a packet *m*. Then, the average aggregation delay is given by:

(19) $$\begin{array}{cc} E[X] & =\sum_{x=1}^{T_{t}}x\cdot P\left[X=x\right]\\ & =\sum_{x=1}^{T_{t}}x\cdot P\left[m\in S_{t-x}|q\left(1\right)>0\right]\\ & =\frac{1}{1-\alpha_{0}}\sum_{x=1}^{T_{t}}x\cdot P\left[m\in S_{t-x},q\left(1\right)>0\right]\\ & =\frac{1}{1-\alpha_{0}}\sum_{t=1}^{T_{t}}\sum_{x=1}^{t}xP\left[m\in S_{t-x},q\left(1\right)>0|PA_{N_{t}}^{T_{t}}=t\right]P\left[PA_{N_{t}}^{T_{t}}=t\right]\\ & =\frac{1}{1-\alpha_{0}}\sum_{t=1}^{T_{t}}P\left[PA_{N_{t}}^{T_{t}}=t\right]\sum_{x=1}^{t}xP\left[m\in S_{t-x},q\left(1\right)>0|PA_{N_{t}}^{T_{t}}=t\right]\\ & =\frac{1}{1-\alpha_{0}}\sum_{t=1}^{T_{t}-1}P\left[PA_{N_{t}}^{T_{t}}=t\right]\sum_{x=1}^{t}xP\left[m\in S_{t-x},q\left(1\right)>0|PA_{N_{t}}^{T_{t}}=t\right]\\ & +\frac{1}{1-\alpha_{0}}P\left[PA_{N_{t}}^{T_{t}}=T_{t}\right]\sum_{x=1}^{T_{t}}xP\left[m\in S_{T_{t}-x},q\left(1\right)>0|PA_{N_{t}}^{T_{t}}=T_{t}\right]. \end{array}$$

The average aggregation delay when the packet aggregation process occurs at time $t<T_{t}$ is decomposed as follows:

(20) $$\begin{array}{cc} \sum_{x=1}^{t}x\cdot P\left[m\in S_{t-x},q\left(1\right)>0|PA_{N_{t}}^{T_{t}}=t\right] & =\;t\cdot P[m\in S_{0},q\left(1\right)>0|PA_{N_{t}}^{T_{t}}=t]\\ & +P[m\in S_{t-1},q\left(1\right)>0|PA_{N_{t}}^{T_{t}}=t]\\ & +\sum_{x=2}^{t-1}x\cdot P\left[m\in S_{t-x},q\left(1\right)>0|PA_{N_{t}}^{T_{t}}=t\right]. \end{array}$$

The event that the packet aggregation process occurs at time $t<T_{t}$ means that there is at least one packet arrival in the first time slot; the number of queued packets at time t−1 is less than $N_{t}$; and the number of queued packets at time *t* is equal to or greater than $N_{t}$. Thus, the probability that the packet *m* arrives at the packet aggregate queue in the first time slot given that the packet aggregation process occurs at time $t<T_{t}$ is given by:

(21) $$\begin{array}{cc} P[m & \in S_{0},q\left(1\right)>0|PA_{N_{t}}^{T_{t}}=t]\\ & =P[m\in S_{0},q\left(1\right)>0|q\left(1\right)>0,N_{t}-C\leq q\left(t-1\right)<N_{t},q\left(t\right)\geq N_{t}]\\ & =\frac{1}{P[PA_{N_{t}}^{T_{t}}=t]}P\left[m\in S_{0},q\left(1\right)>0,N_{t}-C\leq q\left(t-1\right)<N_{t},q\left(t\right)\geq N_{t}\right]\\ & =\frac{1}{P[PA_{N_{t}}^{T_{t}}=t]}\sum_{r=1}^{C}P\left[m\in S_{0},q\left(1\right)=r,N_{t}-r-C\leq q\left(t-1\right)-q\left(1\right)<N_{t}-r,q\left(t\right)\geq N_{t}\right]\alpha_{r}\\ & =\frac{1}{P[PA_{N_{t}}^{T_{t}}=t]}\sum_{r=1}^{C}\sum_{w=N_{t}-r-C}^{N_{t}-r-1}P\left[m\;\in \;S_{0},q\left(1\right)=r,q\left(t\;-\;2\right)=w,q\left(t\right)-q\left(t-1\right)\geq N_{t}\;-\;r\;-\;w\right]\\ & \;\;\;\;\cdot \alpha_{r}{\tilde{p}}_{w}\left(t\;-\;2\right)\\ & =\frac{1}{P[PA_{N_{t}}^{T_{t}}=t]}\sum_{r=1}^{C}\sum_{w=N_{t}-r-C}^{N_{t}-r-1}\sum_{u=N_{t}-r-w}^{C}P\left[m\in S_{0},q\left(1\right)=r,q\left(t-2\right)=w,q\left(1\right)=u\right]\alpha_{r}{\tilde{p}}_{w}\left(t-2\right)\alpha_{u}\\ & =\frac{1}{P[PA_{N_{t}}^{T_{t}}=t]}\sum_{r=1}^{C}\sum_{w=N_{t}-r-C}^{N_{t}-r-1}\sum_{u=N_{t}-r-w}^{C}\frac{r}{u+w+r}\alpha_{r}{\tilde{p}}_{w}\left(t-2\right)\alpha_{u}. \end{array}$$

Similar to Equation (21), the probability that the packet *m* arrives in the packet aggregate queue in time slot [t−1,t] is given by:

(22) $$P\left[m\in S_{t-1},q\left(1\right)>0|PA_{N_{t}}^{T_{t}}=t\right]=\frac{1}{P[PA_{N_{t}}^{T_{t}}=t]}\sum_{r=1}^{C}\sum_{w=N_{t}-r-C}^{N_{t}-r-1}\sum_{u=N_{t}-r-w}^{C}\frac{u}{u+w+r}\alpha_{r}{\tilde{p}}_{w}\left(t-2\right)\alpha_{u}.$$

If the packet *m* does not arrive at the packet aggregate queue during either the time slots [0,1] or [t−1,t], it can be regarded that the packet *m* arrives at the queue uniform randomly during [1,t−1]. The average aggregation delay can therefore be approximated as $\frac{t-1}{2}+1=\frac{t+1}{2}$. Therefore, the average delay in this case is given by:

(23) $$\begin{array}{cc} \sum_{x=2}^{t-1}x\cdot & P[m\in S_{t-x},q\left(1\right)>0,q\left(1\right)>0|PA_{N_{t}}^{T_{t}}=t]\\ & ≅\sum_{x=2}^{t-1}\frac{t+1}{2}\cdot P\left[m\in S_{t-x},q\left(1\right)>0|PA_{N_{t}}^{T_{t}}=t\right]\\ & =\frac{t+1}{2}\sum_{x=2}^{t-1}P\left[m\in S_{t-x},q\left(1\right)>0|PA_{N_{t}}^{T_{t}}=t\right]\\ & =\frac{t+1}{2}\left(1-P[m\in S_{0},q\left(1\right)>0|PA_{N_{t}}^{T_{t}}=t]\right.-P\left[m\in S_{t-1},q\left(1\right)>0|PA_{N_{t}}^{T_{t}}=t\right]\left.\right)\\ & =\frac{t+1}{2}\frac{1}{P[PA_{N_{t}}^{T_{t}}=t]}\sum_{r=1}^{C}\sum_{w=N_{t}-r-C}^{N_{t}-r-1}\sum_{u=N_{t}-r-w}^{C}\frac{w}{u+w+r}\alpha_{r}{\tilde{p}}_{w}\left(t-2\right)\alpha_{u}. \end{array}$$

From Equations (21)–(23), Equation (20) is rewritten as:

(24) $$\begin{array}{cc} \sum_{x=1}^{t}x\cdot & P[m\in S_{t-x},q\left(1\right)>0|PA_{N_{t}}^{T_{t}}=t]\\ & =\frac{1}{P[PA_{N_{t}}^{T_{t}}=t]}\sum_{r=1}^{C}\sum_{w=N_{t}-r-C}^{N_{t}-r-1}\sum_{u=N_{t}-r-w}^{C}\frac{u+\frac{t+1}{2}w+t\cdot r}{u+w+r}\alpha_{r}{\tilde{p}}_{w}\left(t-2\right)\alpha_{u}. \end{array}$$

The packet aggregation process occurrence at time $t=T_{t}$ is inspired by the aggregation timer (i.e., q(1)>0 and $q\left(T_{t}\right)<N_{t}$) or by the packet aggregate threshold (i.e., q(1)>0, $q\left(T_{t}-1\right)<N_{t}$ and $q\left(T_{t}\right)\geq N_{t}$). To describe the above conditions simply, we define new random variable $Z_{N_{t}}^{T_{t}}$ as:

(25) $$Z_{N_{t}}^{T_{t}}=\left\{\begin{array}{cc} 1, & \mathrm{if}\;q\left(1\right)>0,\;N_{t}-C\leq q\left(T_{t}-1\right)<N_{t},\;q\left(T_{t}\right)\geq N_{t}\\ 2, & \mathrm{if}\;q\left(1\right)>0,\;q\left(T_{t}\right)<N_{t}\\ 0, & \mathrm{if}\;q\left(1\right)>0,\;q\left(t\right)\geq N_{t}\;\text{for some}\;t<T_{t} \end{array}.\right.$$

Note that $Z_{N_{t}}^{T_{t}}=0$ means that the packet aggregation process occurs at time $t<T_{t}$ due to the aggregation threshold. The average packet aggregate delay given that the packet aggregation process occurs at time $t=T_{t}$ is decomposed as follows:

(26) $$\begin{array}{cc} \sum_{x=1}^{T_{t}}x & P[m\in S_{T_{t}-x},q\left(1\right)>0|PA_{N_{t}}^{T_{t}}=T_{t}]\\ & =\frac{1}{P[PA_{N_{t}}^{T_{t}}=T_{t}]}\sum_{x=1}^{T_{t}}xP\left[m\in S_{T_{t}-x},q\left(1\right)>0,PA_{N_{t}}^{T_{t}}=T_{t}\right]\\ & =\frac{1}{P[PA_{N_{t}}^{T_{t}}=T_{t}]}\sum_{x=1}^{T_{t}}x\left(P\left[m\in S_{T_{t}-x},q\left(1\right)>0,Z_{N_{t}}^{T_{t}}=1\right]+P\left[m\in S_{T_{t}-x},q\left(1\right)>0,Z_{N_{t}}^{T_{t}}=2\right]\right). \end{array}$$

Using the technique used in Equations (21)–(23), we obtain the following equations:

(27) $$P\left[m\in S_{0},q\left(1\right)>0,Z_{N_{t}}^{T_{t}}=1\right]=\sum_{r=1}^{C}\sum_{w=N_{t}-r-C}^{N_{t}-r-1}\sum_{u=N_{t}-r-w}^{C}\frac{r}{u+w+r}\alpha_{r}{\tilde{p}}_{w}\left(T_{t}-2\right)\alpha_{u},$$

(28) $$P\left[m\in S_{T_{t}-1},q\left(1\right)>0,Z_{N_{t}}^{T_{t}}=1\right]=\sum_{r=1}^{C}\sum_{w=N_{t}-r-C}^{N_{t}-r-1}\sum_{u=N_{t}-r-w}^{C}\frac{u}{u+w+r}\alpha_{r}{\tilde{p}}_{w}\left(T_{t}-2\right)\alpha_{u},$$

(29) $$\begin{array}{cc} \sum_{x=2}^{T_{t}-1}x & P[m\in S_{T_{t}-x},q\left(1\right)>0,Z_{N_{t}}^{T_{t}}=1]\\ & =\frac{T_{t}+1}{2}\left((P\left[Z_{N_{t}}^{T_{t}}=1\right]-P\left[m\in S_{0},Z_{N_{t}}^{T_{t}}=1\right]-P\left[m\in S_{T_{t}-1},Z_{N_{t}}^{T_{t}}=1\right]\right)\\ & =\frac{T_{t}+1}{2}\sum_{r=1}^{C}\sum_{w=N_{t}-r-C}^{N_{t}-r-1}\sum_{u=N_{t}-r-w}^{C}\frac{w}{u+w+r}\alpha_{r}{\tilde{p}}_{w}\left(T_{t}-2\right)\alpha_{u}, \end{array}$$

From Equations (27)–(29), we have:

(30) $$\begin{array}{cc} \sum_{x=1}^{T_{t}}x & P[m\in S_{T_{t}-x},q\left(1\right)>0,Z_{N_{t}}^{T_{t}}=1]\\ & =P\left[m\in S_{T_{t}-1},q\left(1\right)>0,Z_{N_{t}}^{T_{t}}=1\right]+\sum_{x=2}^{T_{t}-1}xP\left[m\in S_{T_{t}-x},q\left(1\right)>0,Z_{N_{t}}^{T_{t}}=1\right]\\ & +T_{t}P\left[m\in S_{0},q\left(1\right)>0,Z_{N_{t}}^{T_{t}}=1\right]\\ & =\sum_{r=1}^{C}\sum_{w=N_{t}-r-C}^{N_{t}-r-1}\sum_{u=N_{t}-r-w}^{C}\frac{T_{t}r+\frac{T_{t}+1}{2}w+u}{u+w+r}\alpha_{r}{\tilde{p}}_{w}\left(T_{t}-2\right)\alpha_{u}. \end{array}$$

The probability that packet *m* arrives in the packet aggregate queue during the first time slot given that the packet aggregation process occurs at time $T_{t}$ due to the packet aggregation timer is as follows:

(31) $$\begin{array}{cc} P[m & \in S_{0},q\left(1\right)>0,Z_{N_{t}}^{T_{t}}=2]\\ & =\sum_{r=1}^{C}P\left[m\in S_{0},q\left(1\right)>0,Z_{N_{t}}^{T_{t}}=2\right]\alpha_{r}\\ & =\sum_{r=1}^{C}\sum_{u=0}^{N_{t}-r-1}P\left[m\in S_{0},q\left(1\right)>0,q\left(1\right)=r,q\left(T_{t}-1\right)=u\right]\alpha_{r}{\tilde{p}}_{u}\left(T_{t}-1\right)\\ & =\sum_{r=1}^{C}\sum_{u=0}^{N_{t}-r-1}\frac{r}{u+r}\alpha_{r}{\tilde{p}}_{u}\left(T_{t}-1\right). \end{array}$$

Similar to the discussion for Equation (23), if packet *m* does not arrive in the packet aggregate queue during the first time slot with the assumption that the packet aggregation process occurs at time $T_{t}$ due to the aggregation timer, it can be regarded that packet *m* arrives in the queue uniform randomly during the time interval $\left[1,T_{t}\right]$. The average aggregation delay may therefore be approximated as $\frac{T_{t}}{2}$. The average delay in this case is given by:

(32) $$\begin{array}{cc} \sum_{x=1}^{T_{t}-1}x\cdot & P[m\in S_{T_{t}-x},q\left(1\right)>0,Z_{N_{t}}^{T_{t}}=2]\\ & ≅\sum_{x=1}^{T_{t}-1}\frac{T_{t}}{2}\cdot P\left[m\in S_{T_{t}-x},q\left(1\right)>0,Z_{N_{t}}^{T_{t}}=2\right]\\ & =\frac{T_{t}}{2}\sum_{x=1}^{T_{t}-1}P\left[m\in S_{T_{t}-x},q\left(1\right)>0,Z_{N_{t}}^{T_{t}}=2\right]\\ & =\frac{T_{t}}{2}\left(P\left[Z_{N_{t}}^{T_{t}}=2\right]-P\left[m\in S_{0},q\left(1\right)>0,Z_{N_{t}}^{T_{t}}=2\right]\right)\\ & =\frac{T_{t}}{2}\sum_{r=1}^{C}\sum_{u=0}^{N_{t}-r-1}\frac{u}{u+r}\alpha_{r}{\tilde{p}}_{u}\left(T_{t}-1\right). \end{array}$$

From Equations (31) and (32), we have:

(33) $$\begin{array}{cc} \sum_{x=1}^{T_{t}}x & P[m\in S_{T_{t}-x},q\left(1\right)>0,Z_{N_{t}}^{T_{t}}=2]\\ & =\;T_{t}P\left[m\in S_{0},q\left(1\right)>0,Z_{N_{t}}^{T_{t}}=2\right]+\sum_{x=1}^{T_{t}-1}xP\left[m\in S_{T_{t}-x},q\left(1\right)>0,Z_{N_{t}}^{T_{t}}=2\right]\\ & =\sum_{r=1}^{C}\sum_{u=0}^{N_{t}-r-1}\frac{\frac{T_{t}}{2}u+T_{t}r}{u+r}\alpha_{r}{\tilde{p}}_{u}\left(T_{t}-1\right). \end{array}$$

Using Equations (30) and (33), Equation (26) is re-written as:

(34) $$\begin{array}{cc} \sum_{x=1}^{T_{t}}x & P[m\in S_{T_{t}-x},q\left(1\right)>0|PA_{N_{t}}^{T_{t}}=T_{t}]\\ & =\frac{1}{P[PA_{N_{t}}^{T_{t}}=T_{t}]}\left(\sum_{r=1}^{C}\sum_{u=0}^{N_{t}-r-1}\frac{\frac{T_{t}}{2}u+T_{t}r}{u+r}\alpha_{r}{\tilde{p}}_{u}\left(T_{t}-1\right)\right.\\ & +\left.\sum_{r=1}^{C}\sum_{w=N_{t}-r-C}^{N_{t}-r-1}\sum_{u=N_{t}-r-w}^{C}\frac{T_{t}r+\frac{T_{t}+1}{2}w+u}{u+w+r}\alpha_{r}{\tilde{p}}_{w}\left(T_{t}-2\right)\alpha_{u}\right). \end{array}$$

Finally, combining Equations (24) and (34) with Equation (19), the average aggregation delay is calculated as follows.

(35) $$\begin{array}{cc} E[X] & =\frac{1}{1-\alpha_{0}}\left(\sum_{r=1}^{C}\sum_{u=0}^{N_{t}-r-1}\frac{\frac{T_{t}}{2}u+T_{t}r}{u+r}\alpha_{r}{\tilde{p}}_{u}\left(T_{t}-1\right)\right.\\ & +\left.\sum_{t=1}^{T_{t}}\sum_{r=1}^{C}\sum_{w=N_{t}-r-C}^{N_{t}-r-1}\sum_{u=N_{t}-r-w}^{C}\frac{u+\frac{t+1}{2}w+t\cdot r}{u+w+r}\alpha_{r}{\tilde{p}}_{w}\left(t-2\right)\alpha_{u}\right). \end{array}$$

## 5. Results and Discussion

The results of the aggregation probability and the average aggregation delay were numerically evaluated as functions of various parameters, such as the value of the aggregation timer, the aggregation threshold, the number of traffic sources and the packet generation probability. We performed Monte Carlo simulations for 100,000 packet arrivals for each scenario in order to validate the numerical analysis. The sensor’s packet generation period is set to 20 ms [22].

**Figure 2:**
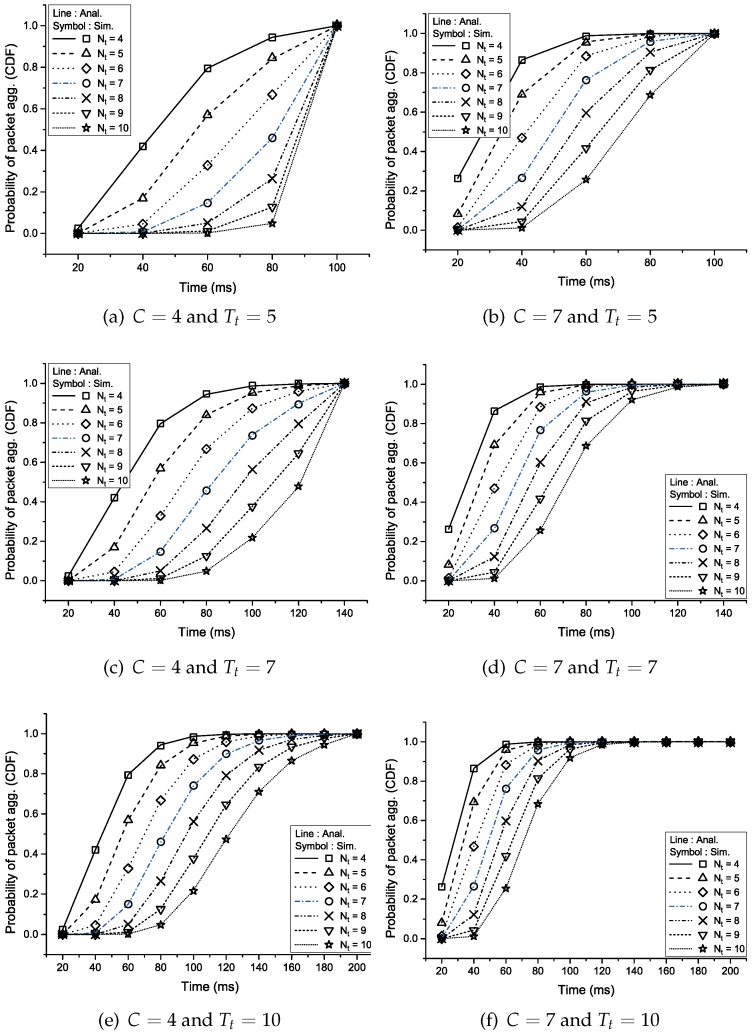
Cumulative distribution function of the aggregation probability.

Figure 2 shows the aggregation probability with respect to the number of traffic sources (*C*), the aggregation timer ($T_{t}$) and the aggregation threshold ($N_{t}$). Naturally, the aggregation probability increases quickly as *C* and $T_{t}$ is greater and $N_{t}$ is smaller. It is shown that the results of the analysis and simulation have good agreement with an error of less than 0.05%.

**Figure 3:**
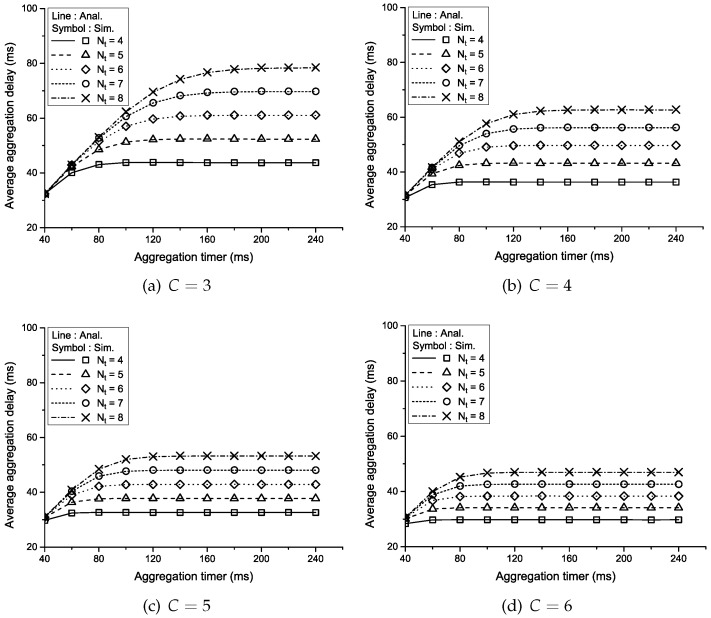
Average aggregation delay vs. the aggregation timer according to $N_{t}$ and *C* when $P_{a}=0.4$.

Figure 3 shows the average aggregation delay versus the aggregation timer according to the aggregation threshold ($N_{t}$) and the number of traffic sources (*C*) when the packet generation probability ($P_{a}$) is 0.4. As the aggregation timer increases, so does the time necessary to generate an aggregated packet, which results in a linear increase in the aggregation delay at the relatively small range of the aggregation timer. However, when the aggregation timer is large enough, most of the aggregated packets are generated by the aggregation threshold, which explains the saturation of the aggregation delay as the aggregation timer increases. Namely, the aggregation delay is bounded below a certain level by some values of the aggregation threshold and the aggregation timer. As the aggregation threshold increases, the time to wait for a packet to be aggregated into a single aggregated frame increases, which results in an increase in the aggregation delay. On the other hand, as the number of traffic sources increases, there are more packet arrivals in the aggregation queue per unit time, which means that the time required to construct an aggregated packet is shorter and the average aggregation delay decreases.

**Figure 4:**
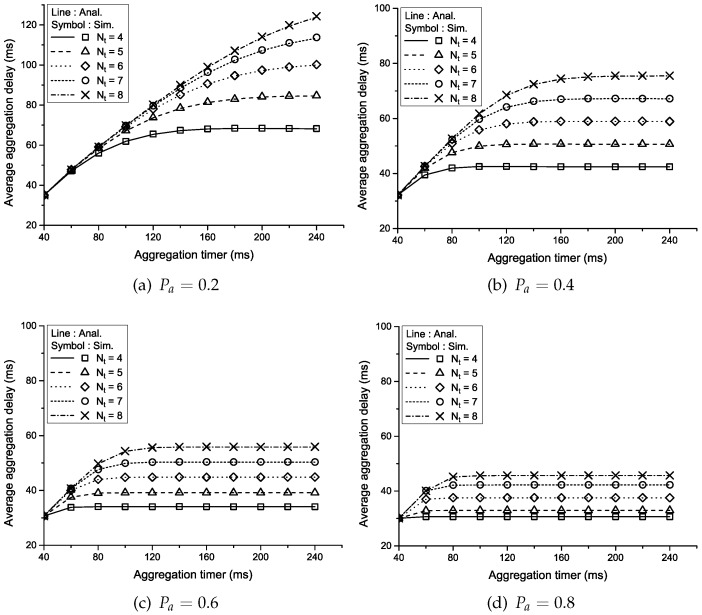
Average aggregation delay vs. the aggregation timer according to $N_{t}$ and $P_{a}$ when C=3.

Figure 4 shows the average aggregation delay versus the aggregation timer according to the aggregation threshold ($N_{t}$) and the probability that a traffic source generates a packet during a unit time ($P_{a}$) when the number of traffic sources (*C*) is three. Similar to the result in Figure 3, the average aggregation delay increases linearly at first as the aggregation timer or aggregation threshold increases and then is saturated at a sufficiently large aggregation timer eventually. In addition, the packet generation probability shows the same effect as the number of traffic sources because more packets arrive at the aggregation queue as $P_{a}$ increases and the time required to construct an aggregated packet is shorter.

## 6. Conclusions

In this paper, we evaluated the delay performance of the packet aggregation process for multisource sensor data with an on-off traffic pattern in the WBAN system. Considering the aggregation timer and the aggregation threshold as dominant parameters that determine the performance of the packet aggregation process, we performed the detailed analysis of the probability that the packet aggregation process occurs during a unit time. Based on the distribution of this probability, the average aggregation delay was formulated in the closed-form. The results showed that the average aggregation delay linearly increases as the aggregation timer or the aggregation threshold increases, but is saturated at sufficiently large values of the aggregation timer or the aggregation threshold, according to the given number of traffic sources and the traffic generation probability. Therefore, this analysis reveals that the additional aggregation delay can be bounded below a certain level by suitably choosing some parameters of the aggregation process, such as the aggregation threshold and the aggregation timer. This means that the considered aggregation threshold and aggregation timer are effective parameters to satisfy the delay requirements of applications. We expect that this study will be able to contribute to provide a theoretical basis to further derive an optimal operational parameter that can maximize the transmission efficiency while guaranteeing the delay constraint of the application in the WBAN system. Moreover, it can provide a general analysis framework for other systems in which the data aggregation occurs and the delay constraint exists, such as the intra-car sensor networks, wireless multimedia sensor networks, mission critical sensor networks, and so on. In the future, we will apply the proposed analysis framework to those sensor networks and try to optimize their performance.

## Figures and Tables

**Table 1:** Definition of the symbols used in the analysis.

Parameter	Definition
*C*	number of traffic sources (i.e., active sensors)
*t*	discrete time index increasing by one per packet generation period
$N_{t}$	packet aggregation threshold
$T_{t}$	value of the packet aggregation timer
$P_{a}$	probability that a traffic source generates sensing data during a packet generation period
$\alpha_{i}$	probability that *i* packets arrive in the queue during the unit time (0≤i≤C)
q(τ)	number of queued packets during the time duration *τ*
$S_{t}$	set of packets arriving in the queue during time interval [t,t+1]
$n_{i}$	the *i*-th packet arriving in the queue
